# Oman, What a Challenge!

**Published:** 1990-09

**Authors:** Michael Whitfield


### From our correspondents

### OMAN—WHAT A CHALLENGE!

Imagine you were put in charge of a country of 2 million inhabitants, most of whom were illiterate, and you were given what seemed to be incredible riches, how would you start to spend the money?

This situation was faced by the present Sultan Qaboos of Oman about twenty years ago and the achievements of the sultanate in the ensuing twenty years have been incredible. The level of literacy has improved dramatically. All children are entitled to free education and there are now about fifty Omani students about to enter their first clinical year in their own medical school at Sultan Qaboos University. I recently spent a week in that medical school observing what is their equivalent of 2nd MB.

Oman is situated along the south-east edge of Arabia and forms the southern land mass of the Straits of Hormuz. It is a Muslim country but considerably more liberal than most others in Arabia. With the oil riches there has been the development of a good road system, a modern airport, fine hospitals and four years ago the formation of the magnificent Sultan Qaboos University. This, in which the medical school is situated, is a few miles away from Muscat, the old walled city and harbour of northern Oman. The country is very 'pro-British'—even the clock tower of the university chimes the identical tones of Big Ben on the hour!

There are roughly equal numbers of male and female medical students and they are taught together—but sit on either side of the room. There are going to be difficult problems ahead for the students who are thoroughly instilled with cultural taboos about touching people of the opposite sex. The problems that will be faced by clinical teachers will be great. My first major gaffe was offering to shake the hand of a fourth year female student. She wrapped her hand in her shawl before accepting my greeting!

Students are taught in English and the medical school is staffed by teachers of many nationalities. Many are British but there is also a strong Scandinavian contingent. The Dean is a Canadian psychiatrist and the professor of Child Health is the Omani minister of health.

The medical school has a strong community based curriculum. The aim is clearly to try to encourage the development of generalists who will, in the future, staff rural clinical and peripheral hospitals. Medical students, many of whom originate from small villages, spend time in every year of their course, in the villages. Each year 'adopts' a village some 10–15 Km from the medical school and, in groups, pursue a different project each term during the four year pre-clinical course. These projects include such titles as 'the six commonest diseases in Fanja', traditional village healers, and so on.

The community based teaching is organised by the Department of Family and Community Medicine (FamCo) which is designed to have equal prominence within the medical school as Anatomy and Surgery. FamCo has the same number of marks to give for assessment of each student at the end of each year as do the traditional disciplines. The planned clinical course is even more radical. Students, in groups of eight, will be attached to FamCo for a period of eight weeks during the first clinical year. They will be placed partly in village clinics and partly in a clinic within the teaching hospital. They will learn clinical method in this setting and something about the management of patients in the community. They will also have a senior FamCo firm in their last clinical year.

What are these village clinics like? Well, they are very variable. I saw two, one of which didnt even keep personal medical records for each patient seen. There was simply an entry of name and diagnosis in the day book. The antenatal and child development records, however, were excellent and the mother kept a carbon copy of the record to bring to each clinic session. In contrast to the magnificent teaching hospital, the structure and support of these village clinics is very poor and will certainly need to be improved if they are to attract the future Omani graduates. At the moment they are a poor contrast to the opulence of the tertiary care centres.

The major problem facing the medical school at the moment is the lack of teachers within the Department of Family and Community Medicine. There is an urgent need for experienced teachers together with doctors who have just completed their vocational training. Why not spend a year or two in Oman rather than opting for the traditional year or two in Australia? You will be reasonably well paid, will be helping an emerging nation and will have incredible opportunities for developing educational and research ideas and projects. There have, for instance, been no community surveys of illness patterns in Oman. If you have any interest in this, please contact me and I will give you more details. What else is there to do in Oman? The countryside is magnificent. There are mountains close to the coast, genuine deserts, green areas in the south of the country, and magnificent unspoilt beaches. There is practically no tourism and ex-patriates spend most weekends exploring the countryside in four-wheel drive vehicles. The degree of honesty within the country and towns is very high. Cars are not locked and stories of forgotten handbags being returned over considerable distances are common. Perhaps this has something to do with the risk of having a hand chopped off if caught stealing. I certainly didn't see any Omani with one hand!

The overall atmosphere (apart from being rather hot in the summer) was one of friendliness and openness. If you are fortunate to get an appointment in that country you will certainly have a wonderful time.

MICHAEL WHITFIELD

